# Supplementary material for: Bioinspired and Post-Functionalized 3D-Printed Surfaces with Parahydrophobic Properties
Source: Biomimetics (Basel). 2021 Dec 13;6(4):71. doi: 10.3390/biomimetics6040071 (PMC8698420; doi:10.3390/biomimetics6040071)
Supplement: Supplementary file 1 [file biomimetics-06-00071-s001.zip › biomimetics-1495438-supplementary.pdf]

Bioinspired and Post-functionalized 3D-printed surfaces for water harvesting (Supplementary data)

Léna Ciffréo [1], Claire Marchand [1], Caroline R. Szczepanski [2], Marie-Gabrielle Medici [3] and Guilhem Godeau [1,3,*]

[1] Institut Méditerranéen du Risque de l'Environnement et du Développement Durable (IMREDD), Université Côte d'Azur, 06200 Nice, France
[2] Department of Chemical Engineering & Materials Science, Michigan State University, East Lansing, MI 48824, USA
[3] Institut de Physique de Nice (INPHYNI), Université Côte d'Azur, UMR 7010, 06000 Nice, France;
* Correspondence: guilhem.godeau@univ-cotedazur.fr; Tel.: +33-489-152-904

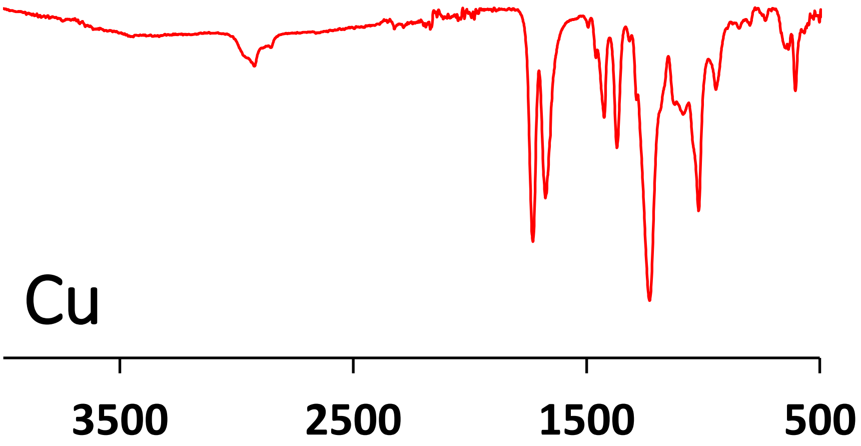

Figure S1. Example of IR for Cu surface.

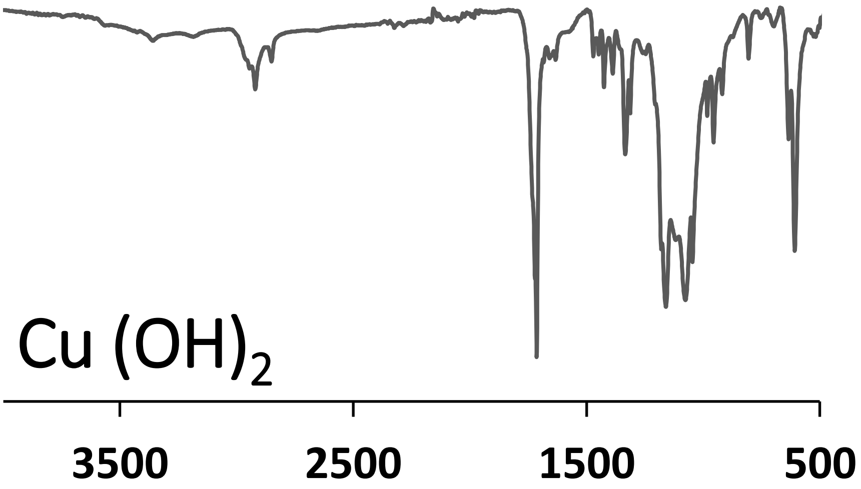

Figure S2. Example of IR for $Cu(OH)_2$ surface.

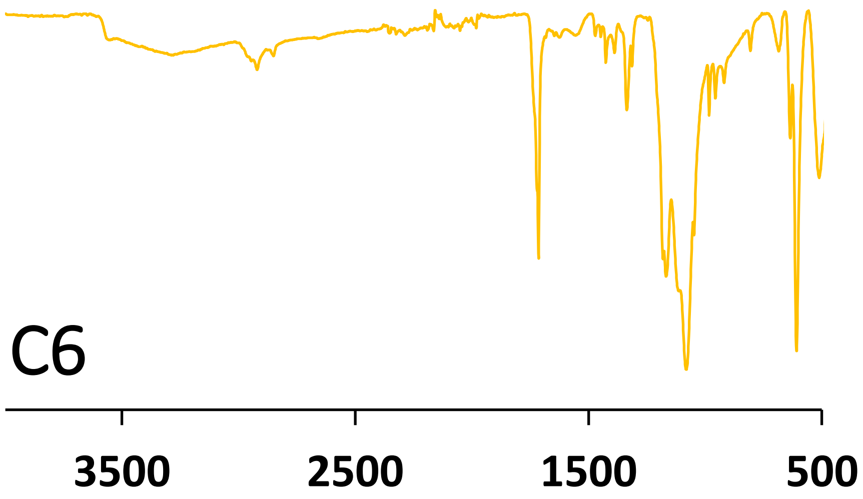

Figure S3. Example of IR for C6 surface.

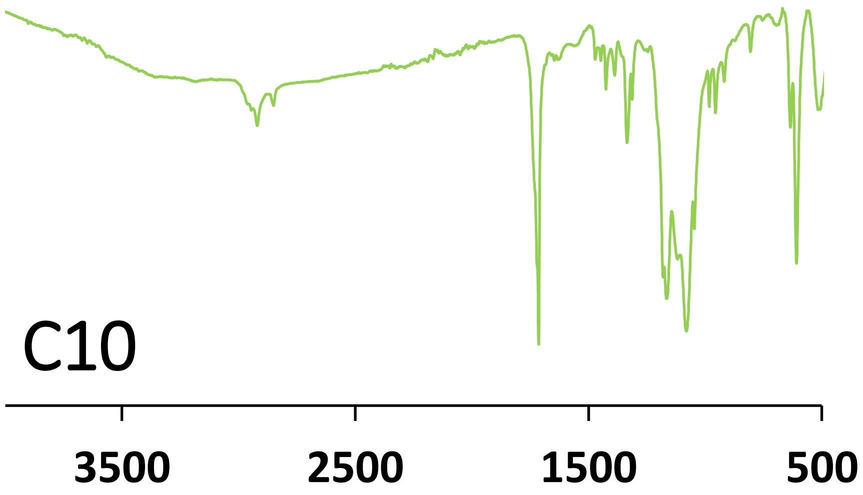

Figure S4. Example of IR for C10 surface.

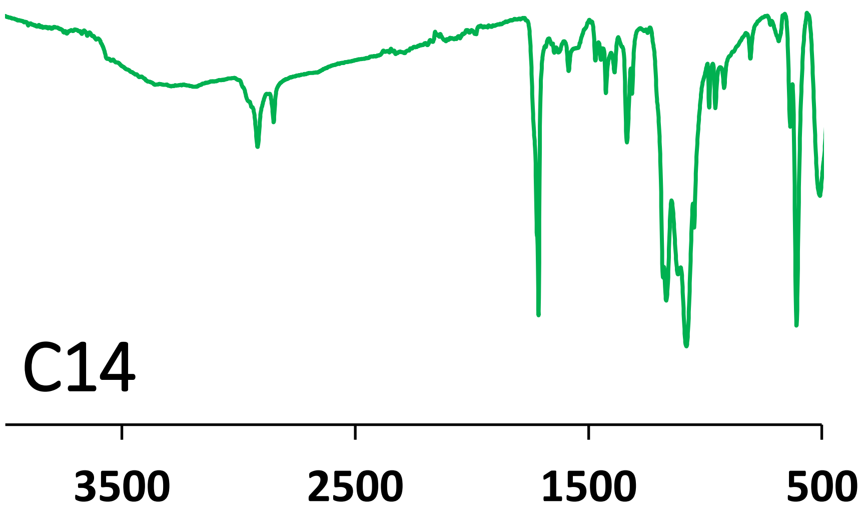

Figure S5. Example of IR for C14 surface.

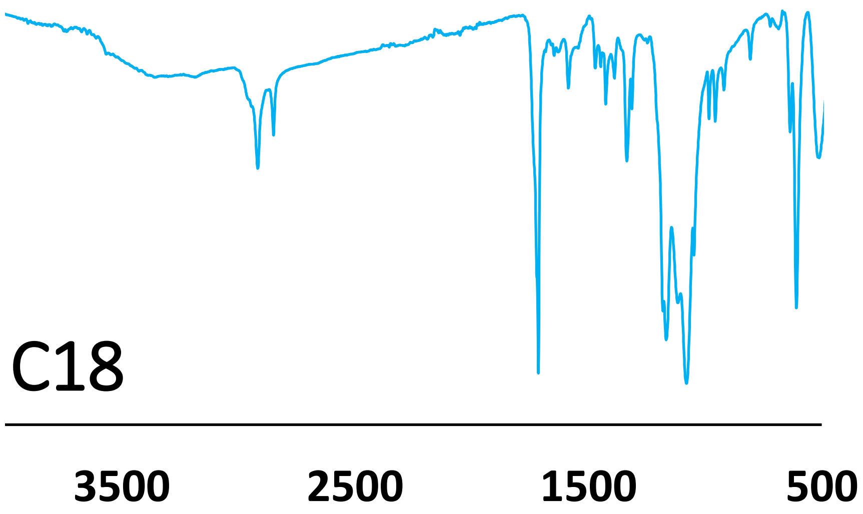

Figure S6. Example of IR for C18 surface.

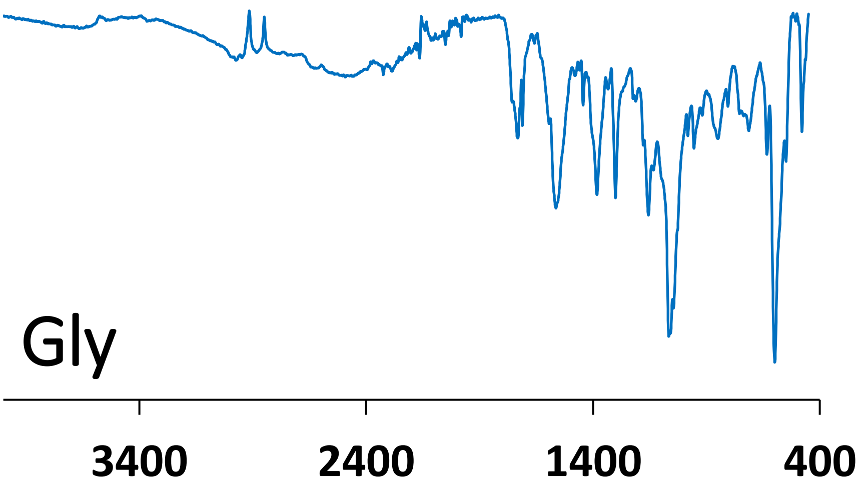

Figure S7. Example of IR for Gly surface.

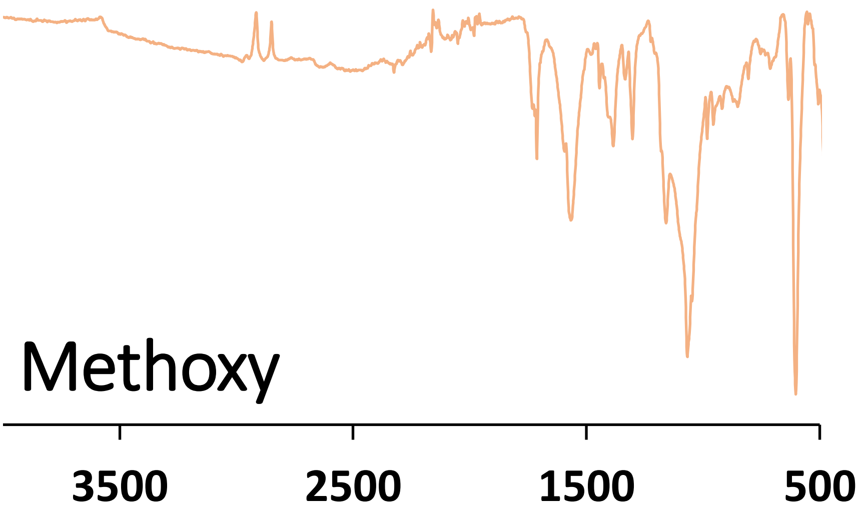

Figure S8. Example of IR for Methoxy surface.

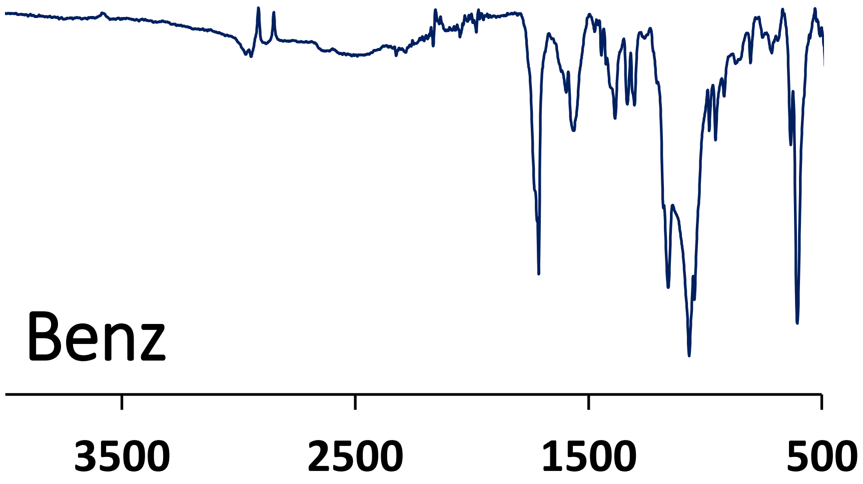

Figure S9. Example of IR for Benz surface.

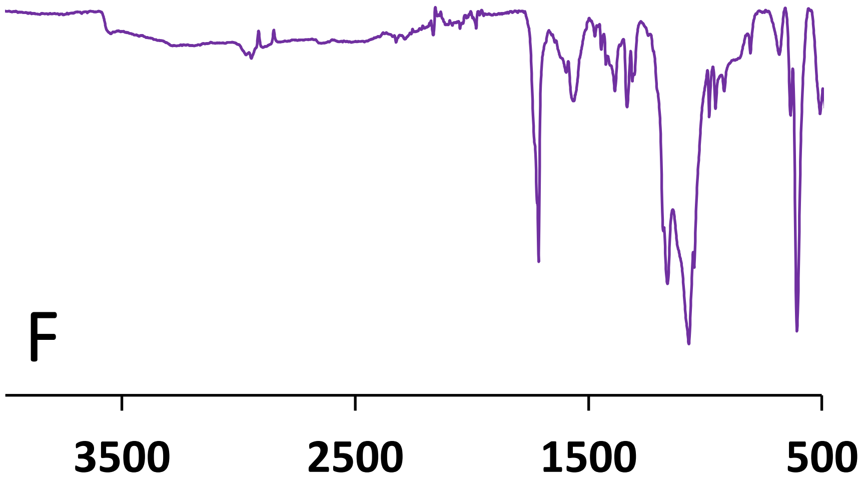

Figure S10. Example of IR for F surface.

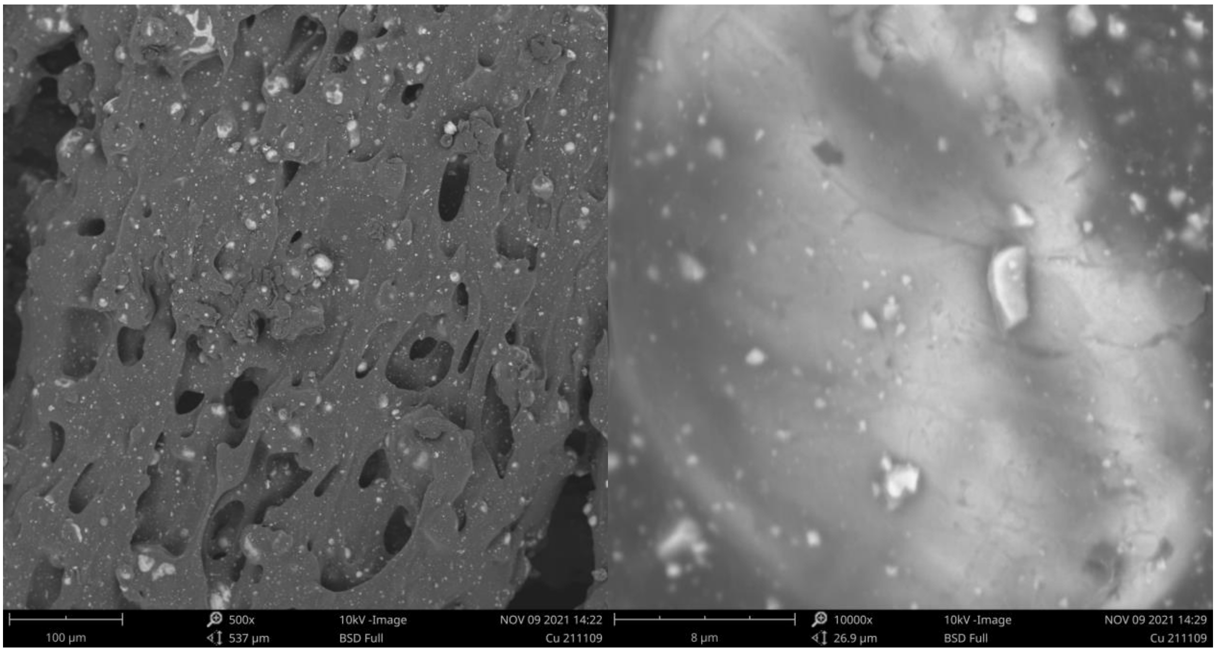

Figure S11. Example of SEM images for Cu surface. (Left: low magnification, Right: high magnification)

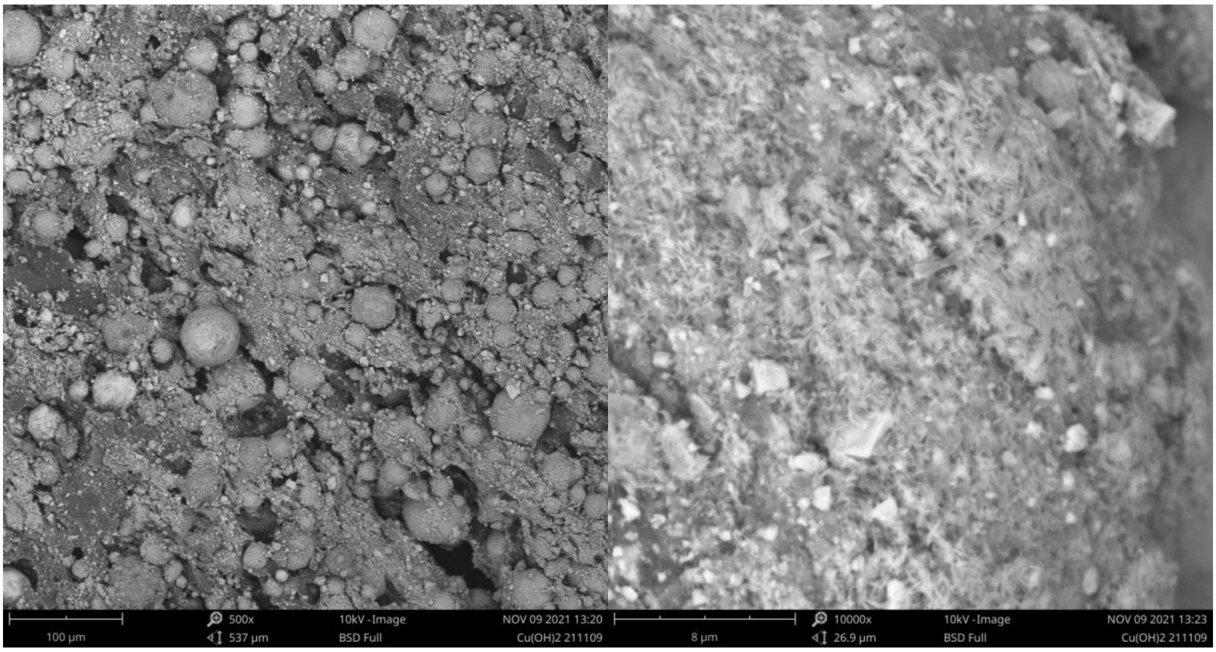

Figure S12. Example of SEM images for $Cu(OH)_2$ surface. (Left: low magnification, Right: high magnification)

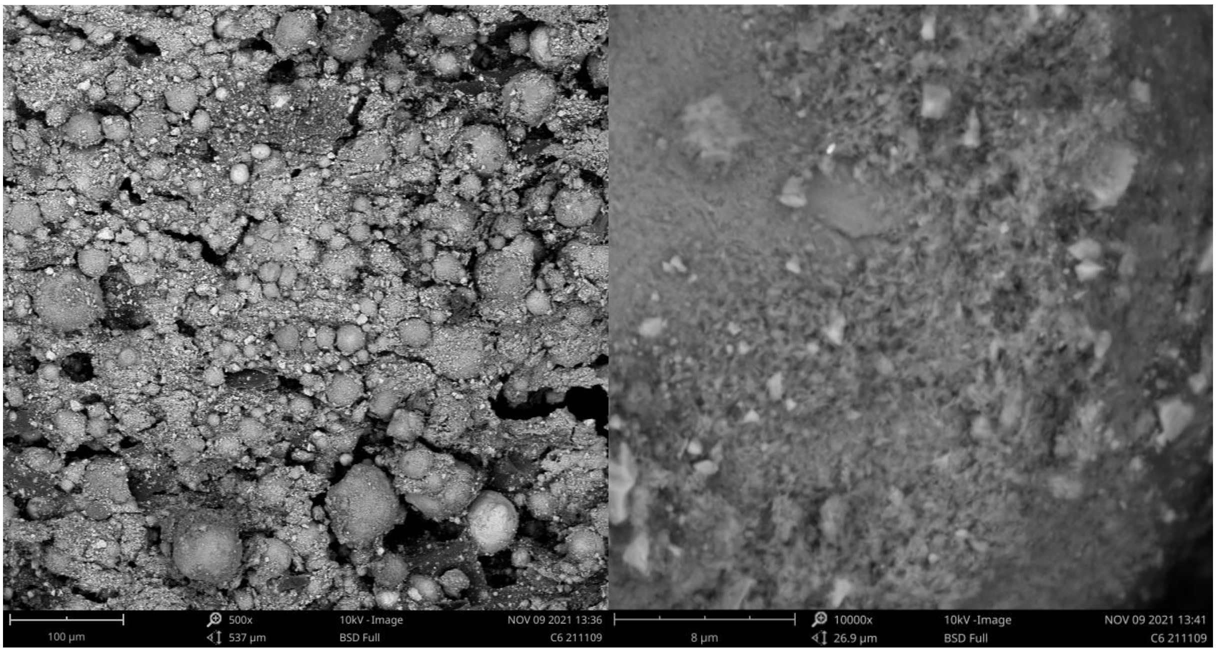

Figure S13. Example of SEM images for C6 surface. (Left: low magnification, Right: high magnification)

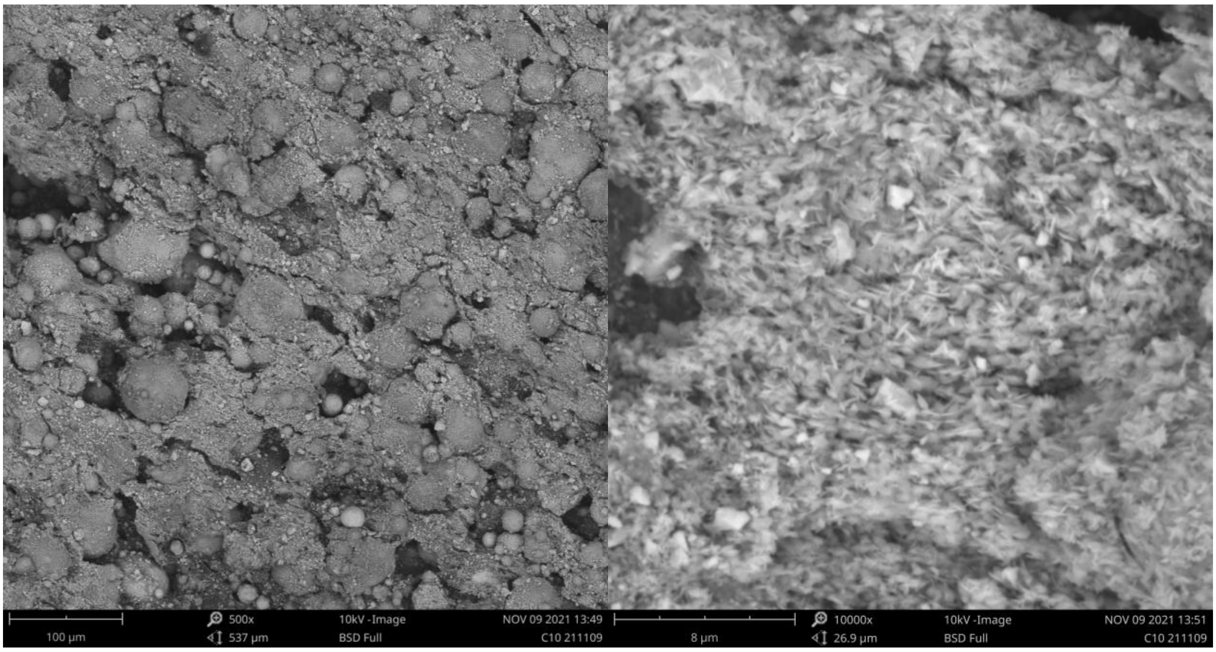

Figure S14. Example of SEM images for C10 surface. (Left: low magnification, Right: high magnification)

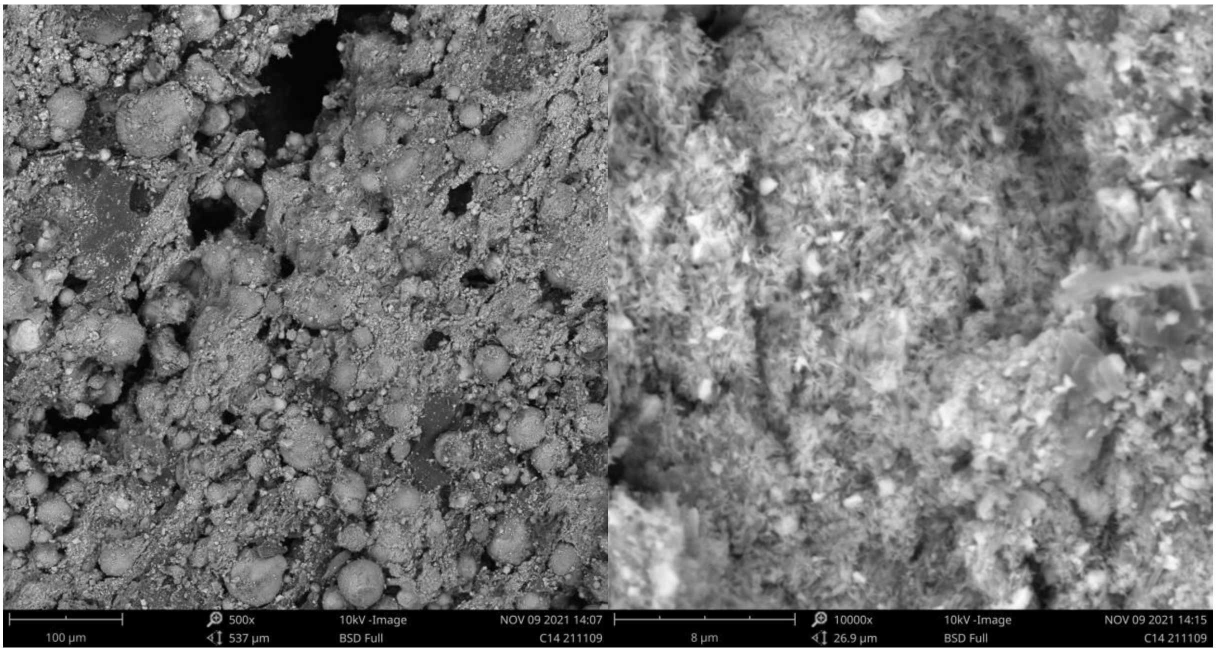

Figure S15. Example of SEM images for C14 surface. (Left: low magnification, Right: high magnification)

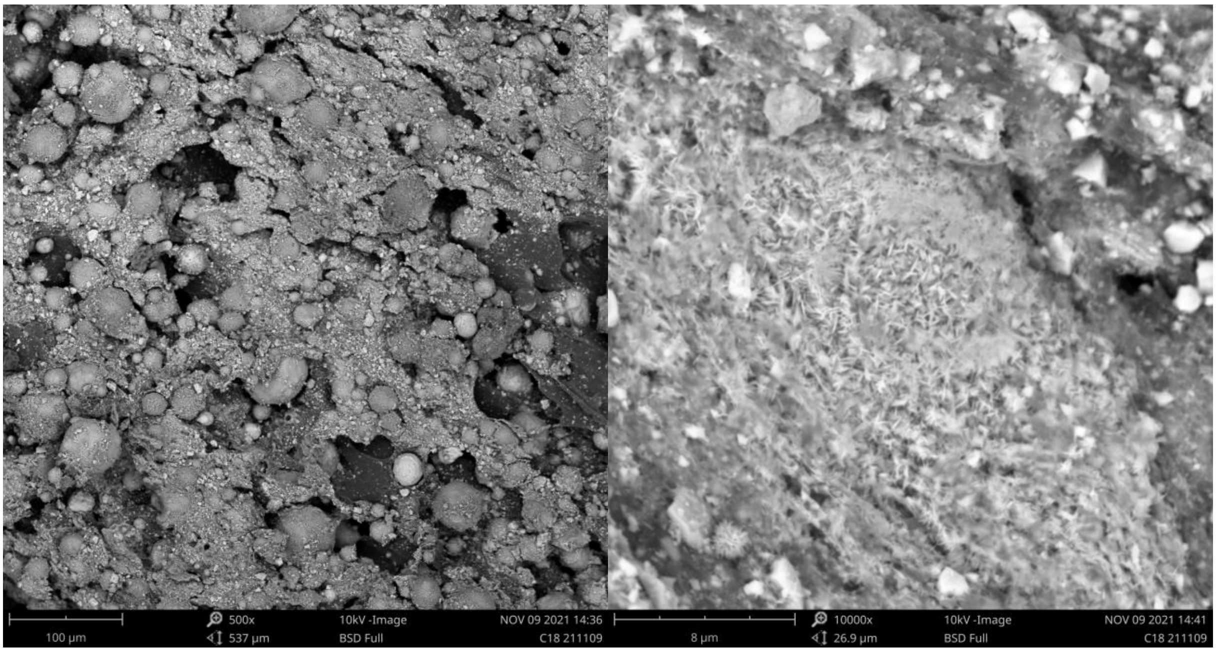

Figure S16. Example of SEM images for C18 surface. (Left: low magnification, Right: high magnification)

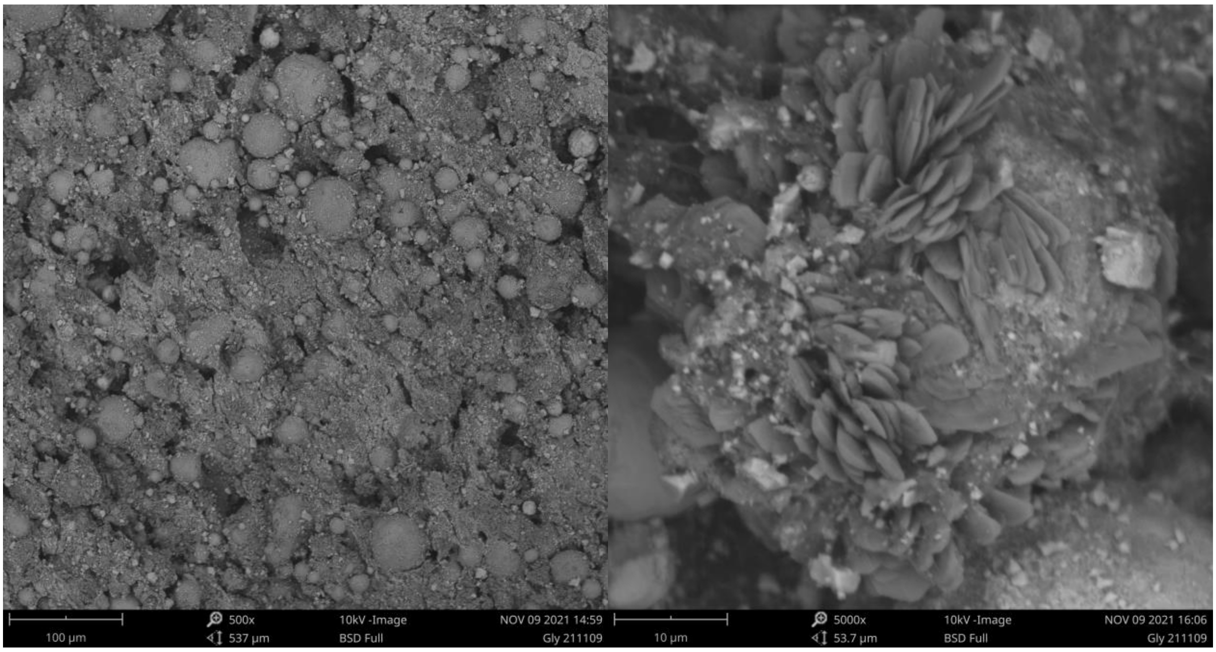

Figure S17. Example of SEM images for Gly surface. (Left: low magnification, Right: high magnification)

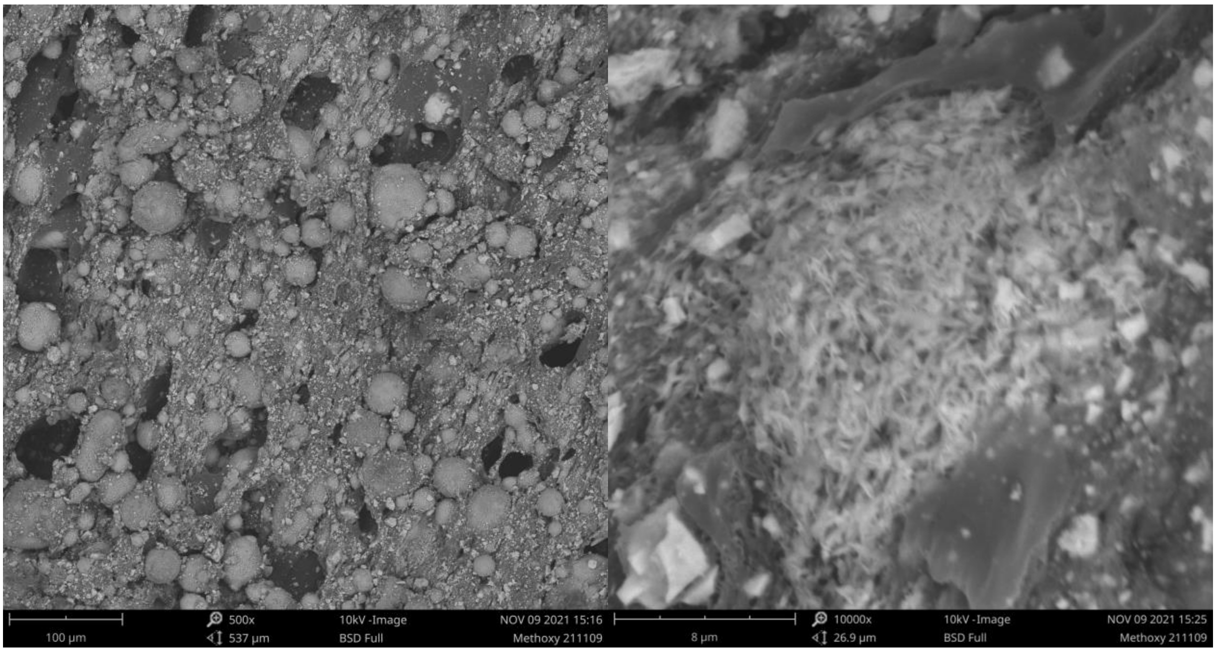

Figure S18. Example of SEM images for Methoxy surface. (Left: low magnification, Right: high magnification)

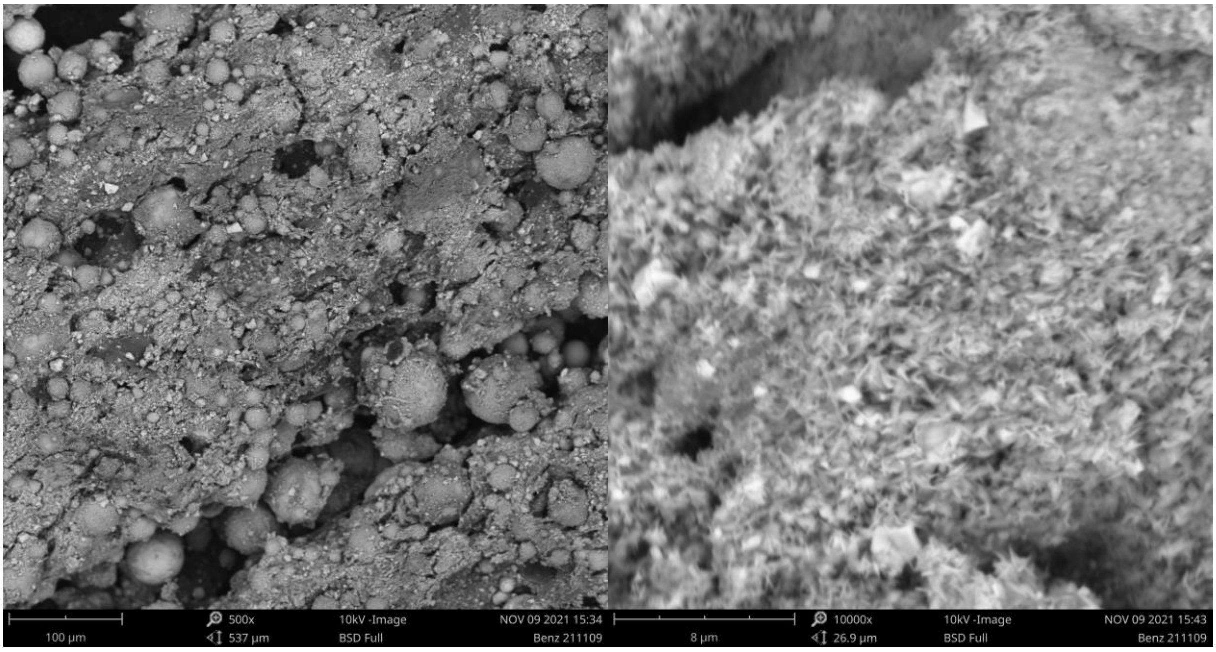

Figure S19. Example of SEM images for Benz surface.

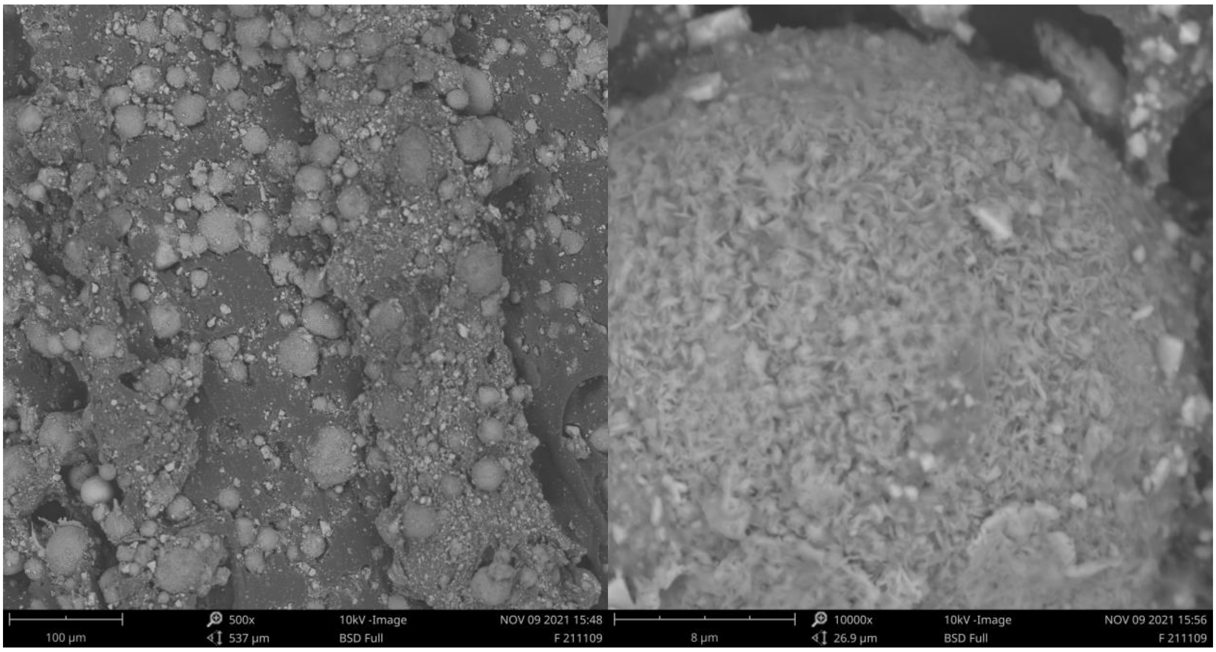

Figure S20. Example of SEM images for F surface.
